# Synthesis of Eu^3+^-Doped NaGd_9_Si_6_O_26_ Sub-Microcrystals from a NaGdF_4_@SiO_2_ Structure

**DOI:** 10.3390/molecules28104214

**Published:** 2023-05-20

**Authors:** Tianyun Du, Xiaojie Xue, Xiuxun Han

**Affiliations:** 1 Institute of Optoelectronic Materials and Devices, Faculty of Materials Metallurgy and Chemistry, Jiangxi University of Science and Technology, Ganzhou 341000, China; 2 Guorui Scientific Innovation Rare Earth Functional Materials (Ganzhou) Co., Ltd., Ganzhou 341000, China; 3 School of Automation Engineering, University of Electronic Science and Technology of China, Chengdu 611731, China; 4 National Rare Earth Function Materials Innovation Center, Ganzhou 341000, China

**Keywords:** rare earth silicate sub-microcrystals, NaGd9Si6O26, low color deviation

## Abstract

Rare earth silicate phosphors of high quantum efficiency with a stable performance are promising materials in the fields of display and illumination. The grain sizes of products synthesized via the conventional solid-state reaction method are usually too large to satisfy the requirements of color cast and extraction efficiency in high-resolution light-emitting devices (LEDs). We designed a synthetic route and successfully fabricated rare earth silicate NaGd_9_Si_6_O_26_ (NGSO) sub-microcrystals with a size ranging from 550 to 1200 nm. The reaction mechanism and optical properties were systematically investigated. The quantum efficiency of Eu^3+^-activated NGSO sub-microcrystals was about 36.6%. The LED encapsulated with these sub-microcrystals showed lower color deviation and higher lumen efficiency and lumen flux compared to that with NGSO fabricated using the conventional solid state reaction method.

## 1. Introduction

In recent years, europium-doped silicate phosphors have been reported to exhibit high quantum efficiencies and a good structural thermal stability, which have considerable potential for display and illumination [1,2,3]. A conventional method for synthesizing silicate phosphors uses a high-temperature solid-state reaction method, and the particle sizes of the final products are usually large, remaining in the tens of micrometers even after they are ball milled [4,5,6]. Many investigations have demonstrated that the naturally large size of phosphors would lead to less scattering of excitation light within the phosphors, reduce light extraction efficiency, and cause poor angular color uniformity of the device, for example, the “yellow ring” effect in YAG:Ce^3+^-packaged light emitting diodes (LEDs) [7,8,9,10]. With the increasing demand for high-resolution display panels that are composed of lower-sized pixels, such as mini-LEDs and micro-LEDs, the color cast and pixel size mismatch brought by large-sized phosphors will be more severe [11,12]. However, the quantum efficiency of phosphors decreases as the particle size decreases [13]. It is important to select the appropriate size of phosphor for different LEDs. There should be an optimal particle size to balance the increasing extraction efficiency and decreasing quantum efficiency.

Unfortunately, fabrication of rare earth silicate phosphors with a nanoscale size is still a challenge, not to mention the controllable synthesis of various particle sizes. The conventional solid-state reaction (SSR) method requires a high temperature, usually more than 1000 °C, and is impacted by the inhomogeneity of mass and heat transfer, and the sizes of the produced crystals might not be controlled. The samples fabricated using the co-precipitation and sol–gel methods suffer from agglomeration during the high-temperature calcination processes [14]. In comparison to the other methods listed above, the spray pyrolysis method produces rare earth silicate particles with a smaller size distribution, ranging from nanometers to micrometers [15,16]. However, preparing fine and homogeneous droplets that affect the size distribution of the final products and the scaling-up remain difficult [17]. The key to achieving the synthesis of nanomaterials using the bottom-up approach is the isolation between products. Ligand-assistant and space-confined solid state reaction (ScSSR) strategies are commonly used to obtain nanocrystals [18,19]. However, there are no suitable ligands that can maintain the structural stability at such high reaction temperatures for silicate reactions. The synthetic strategy of the ScSSR method requires nanoscale-sized precursors to be coated with an inert nanoshell that ensures that the entire reaction occurs inside the cavity enclosed by the nanoshell, and there is no reaction between the nanoshell and the precursors. Silica has been widely used as an inert nanoshell to synthesize noble metal nanoparticles [20,21,22]. Based on our previous work, the silica-coated NaYF_4_ nanocrystals were proved to be a feasible precursor to form Er^3+^-Yb^3+^-doped NaYSiO_4_ [23]. It implies the probability of preparing other promising rare earth silicates using similar methods, for example, sodium gadolinium silicates. As reported, Eu^3+^ -activated Na_3_GdSi_2_O_7_ exhibited a more pure red emission than commercial phosphor YGB:Eu, which could help improve the color gamut of display devices [24]. The quantum efficiency of Eu^3+^-doped NaGdSiO_4_ was reported to be about 85% in bulk materials [5].

Here, we report the successful synthesis of NaGd_9_Si_6_O_26_ sub-microcrystals (sub-MCs) using an ScSSR-like method. The sub-MCs were rod-shaped with an average size of about 550 nm. The structure and morphology of the samples at various stages of the fabrication process were investigated to clarify the mechanism of oriented growth in the high-temperature reactions. The luminescent properties of 50 mol% of Eu^3+^-doped NaGd_9_Si_6_O_26_ sub-MCs and microcrystals (MCs prepared using the conventional high-temperature solid-state reaction) were also investigated. Their quantum efficiency was about 36.6 and 91.5%, respectively, for sub-MCs and MC materials, which might have a high value, if ever published. The Eu^3+^-doped NaGd_9_Si_6_O_26_ sub-MCs were packaged with a 395 nm LED that shows a high angular color uniformity and enhanced luminous efficiency.

## 2. Results and Discussion

**Figure 1:**
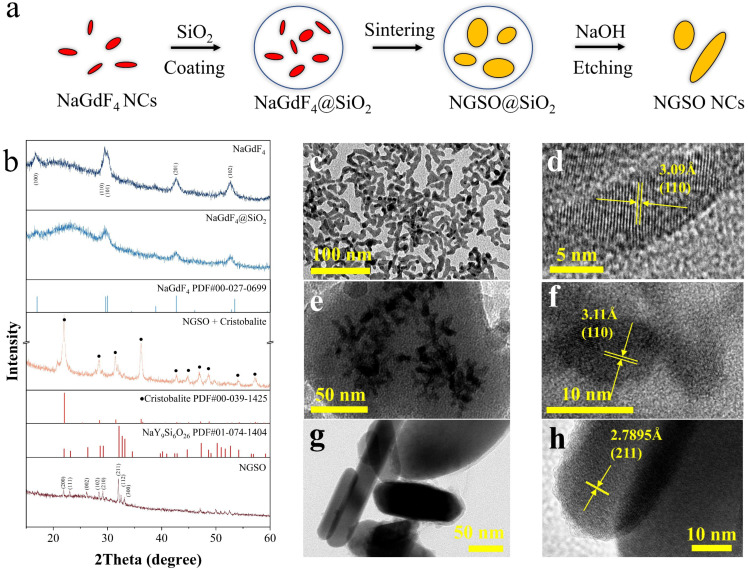
(**a**) A schematic diagram of the synthetic procedure. (**b**) XRD patterns of NaGdF_4_, NaGdF_4_@SiO_2_, NGSO@SiO_2_, NGSO, and TEM, and HRTEM images of NaGdF_4_ (**c**,**d**), NaGdF_4_@SiO_2_ (**e**,**f**), and NGSO (**g**,**h**), respectively.

We designed a synthetic procedure that uses core/shell nanoparticles as reaction units and that etches excessive silica to obtain pure NaGd_9_Si_6_O_26_ sub-MCs. A schematic diagram of the experimental process is shown in Figure 1a.

The XRD patterns in Figure 1b reveal the phase of products at different stages. The diffraction peaks in the XRD pattern of the NaGdF_4_ sample match well with those from the standard phase NaGdF_4_ (PDF#00-027-0699) [25,26]. It indicates that the as-prepared sample consisted of pure NaGdF_4_ crystals with a hexagonal phase. From the TEM images in Figure 1c, it can be observed that the NaGdF_4_ sample synthesized via a solvothermal method was rod-shaped. The average size was 8.5 and 31 nm in diameter and length, respectively. A crystal lattice can be observed clearly in Figure 1d. The spacing of the lattice fringe was measured to be about 3.09 Å, which is assigned to the (110) facet of the hexagonal phase of NaGdF_4_.

The diffraction pattern of the NaGdF_4_@SiO_2_ nanoparticles still matched with NaGdF_4_ (PDF#00-027-0699), but the intensity was relatively reduced. Additionally, a broad band of amorphous silica appeared at nearly 23 °C [27,28]. The TEM image of the NaGdF_4_@SiO_2_ sample in Figure 1e shows a clear core/shell structure. Lattice fringes could be observed in the dark parts of the HRTEM image shown in Figure 1f. The spacing of the fringe is about 3.11 Å which is also assigned to the (110) facet of the hexagonal phase NaGdF_4_. No lattice fringes could be found in the gray parts of the TEM images, which indicates that they were amorphous SiO_2_. The big particle of about 200 nm in Figure 1e reveals that NaGdF_4_ nanocrystals were enclosed by SiO_2_ coating with a thickness of about 30 nm. The small completely gray circles about 20 nm were pure SiO_2_ nanoparticles without a NaGdF_4_ core inside. It confirms the existence of silica and the silica coating procedure did not affect the morphology and crystal structure of the NaGdF_4_ core. Following sintering, the core/shell nanoparticles reacted and were transferred to new phases.

The diffraction peaks centered at 22 °C, 28.4 °C, 31.4 °C, and 36.2 °C could be assigned to the (101), (111), (102), and (200) facets of cristobalite (PDF#00-089-3434). It implies that a phase transition process from amorphous silica to cristobalite occurred. While the peaks at 29 °C, 31.9 °C, 32.3 °C, and 33.1 °C did not match well with any standard PDF cards, the profile of those peaks was close to the NaY_9_Si_6_O_26_ (PDF#01-074-1404) of the oxyapatite structure [29]. Since rare earth ions have similar physical and chemical properties, Y^3+^ might be substituted with Gd^3+^ in the cells. The larger radius of Gd^3+^ would cause diffraction peak positions to shift in the direction towards a small angle. It can be asserted that the as-prepared sample contained NaGd_9_Si_6_O_26_, denoted as NGSO for simplicity.

The diffraction peaks in the XRD pattern of the etched sample matched well with those of pure NaY_9_Si_6_O_26_, and no contribution of cristobalite was observed. It illustrates that the excessive cristobalite was completely etched via an alkaline etching process, and the final product was the pure hexagonal phase NGSO. The structure of the as-synthesized NGSO was refined using the Rietveld method from a CIF file of hexagonal phase NaY_9_Si_6_O_26_, and the XRD patterns calculated after the refinement structure are shown in Figure S1. The table of the lattice parameters is shown in Table S2. The value of R${}_{wp}$ and $\chi^{2}$ was 4.576 and 1.62, indicating that the diffraction peaks of the corrected crystal structure matched well with the experimental data. The results showed that the phase and space group of NGSO was hexagonal and P6_3_/m was the same with NaY_9_Si_6_O_26_. The length of a and b increased from 9.334 Å to 9.361 Å, and the volume of the cell increased simultaneously from 509.98 Å^3^ to 516.84 Å^3^. Owing to the larger-sized Gd atoms replacing Y atoms, the lattice parameters were expanded. The TEM images in Figure 1g–h identify the crystallinity of the as-synthesized NGSO as good. The fringe spacing was about 2.81 Å corresponding to the (211) facet. The morphology of the etched sample consisted of nanorods with 114 nm in length and 25 nm in diameter. Interestingly, the average size of NGSO nanorods was much larger than the size of NaGdF_4_@SiO_2_ nanoparticles. We speculated that this may be due to the aggregation of NGSO NCs and the oriented growth under high temperatures. Because the sintering temperature is lower than the melting point of crystalline silica, it is quite difficult for melting to occur on the contact surface of the two adjacent particles and cause further agglomeration. According to the proposed reaction mechanism between NaYF_4_ and SiO_2_ in our previous work, SiF_4_, as one of the main products of the reaction, was released in the form of gas [23]. The melted NGSO may flow through the channels created when the SiF_4_ gas exits the protective layer of silica. The other speculation is the large-sized NGSO was generated by the migration and aggregation of Na^+^ and Gd^3+^ ions due to the reduction of interface energy in the system.

**Figure 2:**
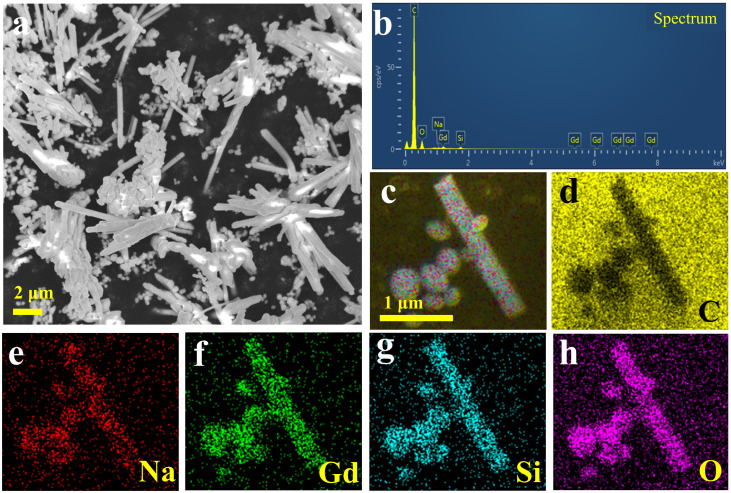
(**a**) SEM images, (**b**) EDS, (**c**) mapping of each element in the NGSO, and (**d**–**h**) distributions of C, Na, Gd, Si, O elements, respectively.

The morphology of the etched NGSO showed it to be rod-shaped with 7 μm in diameter and 450 nm in length, and it contained many ellipsoid grains with a size of hundreds of nanometers, as shown in Figure 2a,b. The EDS mapping in Figure 2c,d clearly shows that the as-prepared sample was composed of Na, Gd, Si, and O elements. The ratio between elements was very close to the stoichiometric ratio of NaGd_9_Si_6_O_26_ (Table S1). It confirms the existence of NaGd_9_Si_6_O_26_, although the standard cards of NaGdSiO^4^ and NaGd_9_Si_6_O_26_ have similar profiles.

**Figure 3:**
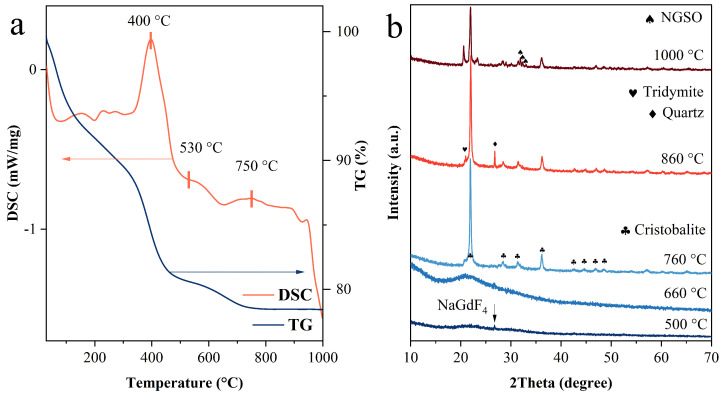
(**a**) TG and DSC curves of NaGdF_4_@SiO_2_ sintered in the range from room temperature to 1000 °C and (**b**) XRD patterns of NaGdF_4_@SiO_2_ sintered at different temperatures.

Compared with the traditional solid-state reaction, the synthetic procedure we developed based on a heterogeneous core/shell structure did not require a higher temperature to supply the reaction due to the high surface energy of nanoparticles [30]. The silicate materials synthesized via a traditional solid-state reaction are usually irregular-shaped bulk materials. However, the existence of rod-shaped grains confirmed the oriented growth in the solid-state reaction we designed. In order to investigate the thermodynamics process of the reaction to the formation of NGSO, we conducted TG and DSC tests in the range from room temperature to 1000 °C at 10 °C/min, as shown in Figure 3. The TG curve showed a rapid decline before 100 °C, which indicated the evaporation of adsorbed water molecules. The weight loss in the range of 330–450 °C was attributed to the combustion of residual organic matter such as oleic acid or CO-520 molecules. In the same range of temperature, the DSC curve showed an exothermic peak that was consistent with the analysis of the TG curve. There was an endothermic peak in the range of 530–750 °C, and the weight of the sample slowly decreased as the temperature increased. When the reaction temperature was higher than 750 °C, the weight of the sample remained stable. From the XRD pattern of the sample sintered at 500 °C in Figure 3b, a weak diffraction peak could be observed, which was assigned to the (110) facet of NaGdF_4_. As the temperature further increased to 660 °C, the diffraction peak from NaGdF_4_ disappeared, and only a broad band attributed to amorphous SiO_2_ was left. It was speculated that crystalline NaGdF_4_ may have been dispersed and replaced with the presence of amorphous NaGdF_4_, according to the results we observed in the previous study [23]. When the sintering temperature increased to 760 °C, all of the amorphous SiO_2_ and NaGdF_4_ were converted to cristobalite and NGSO, respectively. It was confirmed by the existence of the single endothermic peak in the DSC curve between 530 °C and 750 °C in Figure 3a. It is worth noting that the amorphous silica transformed directly into cristobalite instead of quartz, which is the thermodynamically stable phase in this temperature range. One explanation for this phenomenon is that there exists some part of the pre-crystalline structure of cristobalite in amorphous silica, as well as some short range disorders in cristobalites [31].

With the temperature further increased to 860 °C, the cristobalite phase partly transformed into quartz and tridymite phases. This process was fully completed when the temperature reached 1000 °C. In addition to the phase transition process of silica, the intensity of the diffraction peaks of the NGSO phase also gradually increased. It indicates that the reaction was more adequate. Since the DSC peak of the reaction between NaGdF_4_ and SiO_2_ was covered by the phase transition of SiO_2_, it was not easy to estimate the specific value of the activation energy using the Kissinger method.

**Figure 4:**
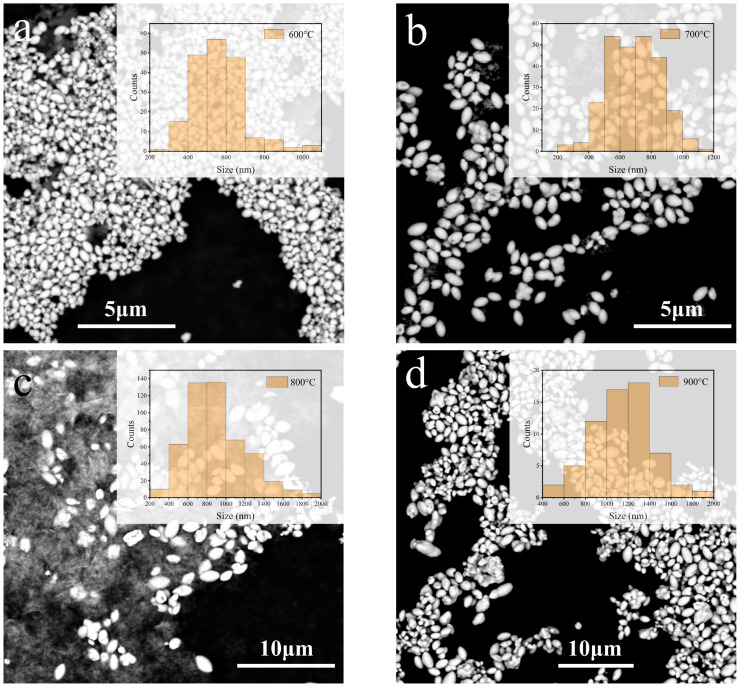
Size of NGSO nanoparticles sintered at different temperatures: (**a**) 600 °C, (**b**) 700 °C, (**c**) 800 °C, and (**d**) 900 °C.

The influence of heating conditions on the morphology was investigated using core-shell nanoparticles sintered at various temperatures. Although the heating temperature was different, all samples were presented as dispersed particles, as shown in Figure 4. Unlike sol–gel or chemical co-precipitation methods, the synthesis scheme designed in this paper can effectively avoid the aggregation of rare earth silicate particles. As the temperature increased from 600 to 900 °C, the average grain size of the NGSO increased from 500 to 1200 nm. It implies that higher temperatures lead to the fast growth of NGSO particles.

**Figure 5:**
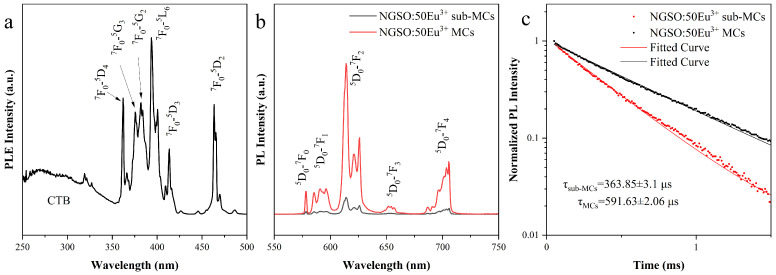
PLE (**a**), PL (**b**), and emission decay curve (**c**) at 614 nm of NGSO:50Eu^3+^ sub-MCs and MCs.

Recently, 50 mol% Eu^3+^-doped luminescent materials of high quantum efficiency have been reported [1,5]. We have successfully fabricated 50 mol% Eu^3+^-doped NGSO sub-MC and MC materials. Their XRD patterns were consistent with NaY_9_Si_6_O_26_ (PDF#01-074-1404). The structure and morphology data are shown in Figures S2–S6. The PLE spectrum of NGSO:50Eu^3+^ sub-MCs monitored at 614 nm is shown in Figure 5a, which is the radiative transition of ^5^D_0_→^7^F_2_. The broadband with a relatively low intensity centered at around 270 nm might be assigned to the charge transfer band (CTB) from Eu^3+^ to O^2−^ [32]. Other excitation peaks were associated with transitions from the ground state of Eu^3+^ to high-lying energy levels with a 4f configuration. The transitions of Eu^3+^ at 360, 375, 381, 394, 413, and 463 nm ^7^F_0_→^5^D_4_, ^7^F_0_→^5^G_3_, ^7^F_0_→^5^G_2_, ^7^F_0_→^5^L_6_, ^7^F_0_→^5^D_3_, and ^7^F_0_→^5^D_2_ were observed in the range of 350–500 nm. Figure 5b shows the PL spectra of Eu^3+^-doped sub-MC and MC materials. Under the excitation of 394 nm light, the samples show intense red light emissions. The emission peaks located between 550 and 600 nm originated from the ^5^D_0_→^7^F_J_ (J = 0, 1) transition. The emission near 656 nm corresponded to the ^5^D_0_→^7^F_3_ transition. The strongest emission peaks around 614 nm were assigned to the ^5^D_0_→^7^F_2_ transition. Other emission peaks near 700 nm came from the ^5^D_0_→^7^F_4_ transition. All emission peaks were generated from the f-f transition of Eu^3+^. The intensity of the emission from the NGSO:50Eu^3+^MCs sample was much stronger than the sub-MCs. The quantitative investigation was carried out with the measurement of the photoluminescence quantum yield (PLQY). The PLQY of NGSO:50Eu^3+^ MCs was prepared using the high-temperature solid-state reaction up to 91.5%, while that of the as-prepared sub-MCs was about 36.6% (shown in Figures S7 and S8). It is worth mentioning that 91.5% is an extremely high value for Eu^3+^ doping. Compared with that, the reduction of PLQY in sub-MCs might be caused by strong nonradiative transitions from surface defects due to the high surface-to-volume ratio in nanomaterials [13].

Figure 5c exhibits the decay curves of emissions at 614 nm in NGSO:50Eu^3+^ sub-MCs and MCs under excitation from 394 nm. The decay curves were well fitted using a single exponential equation: [33]

(1) $$I\left(t\right)=I_{0}+Aexp\left(-t/\tau \right)$$

where I(t) is the photoluminescence intensity at time *t*. $I_{0}$, A is the constant, and τ is the photoluminescence lifetime. The obtained lifetimes of the ^5^D_0_ energy level for the ^5^D_0_→^7^F_2_ are 363.9 and 591.6 μs in NGSO:50Eu^3+^ sub-MCs and MCs, respectively. The decline in the PLQY is consistent with the tendency of the emission lifetime in those two samples.

We encapsulated the as-prepared NGSO:50Eu^3+^ sub-MCs and MCs in LED chips, respectively, to test the luminescent characteristics as phosphors. The output power of the LED chips was about 1 W, and the central emission wavelength was about 395 nm. In order to verify the luminescence performance at high density filling, the as-prepared NGSO:Eu^3+^ phosphors were filled into the grooves and compacted in a conformal package.

**Figure 6:**
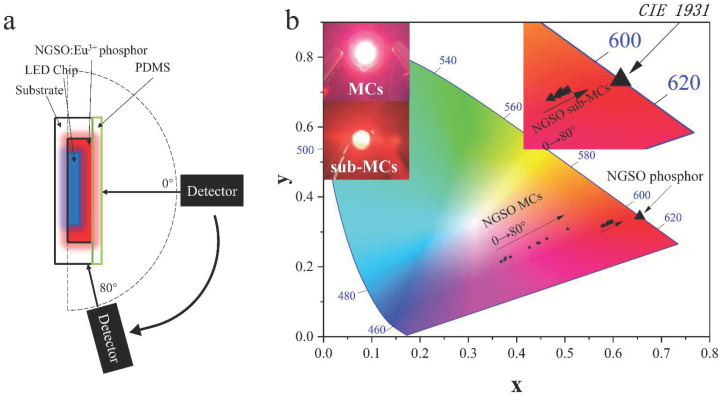
ACU detection method (**a**) and color coordinates in CIE 1931 (**b**).

The angular color uniformity (ACU) is a key property of LEDs. A high ACU is required for illumination products. We designed an apparatus equipped with a spectroscope to acquire the ACU, as shown in Figure 6a. The detector was utilized to measure the emission spectrum from the illuminated LED with phosphors at various angles from 0° to 80°. The color coordinates of sub-MCs and MCs dependent on angles were calculated and marked out in a chromaticity diagram shown in Figure 6b. The star and triangle symbols represent the color coordinates of NGSO MCs and NGSO sub-MCs at different angles, respectively. The coordinates of the LED filled with NGSO:Eu^3+^ MC samples started from the magenta region and shift to the red region. However, those of NGSO:Eu^3+^ sub-MC-filled LED stayed relatively stable in the red region of the chromaticity diagram. The inset images in Figure 6b are the photographs of the NGSO:Eu^3+^ sub-MCs and MC-filled LEDs, respectively. It was obvious that the visual color of the LED for sub-MCs was pure red and that for MCs was magenta. It indicates that the sub-MC-filled LED should have a much better ACU. The ACU could be estimated according to the color deviation calculated using the following equation,

(2) $$\Delta E=\sqrt[{}]{{\left(u_{80}-u_{0}\right)}^{2}+{\left(v_{80}-v_{0}\right)}^{2}+{\left(w_{80}-w_{0}\right)}^{2}},$$

where u=4x/(3−2x+12y), v=9y/(3−2x+12y), w=1−u−v. The value of *x* and *y* presents the color coordinate. The subscript numbers 0 and 80 stand for the value of the detecting angle. The color deviation calculated from Equation (2) of the LED fabricated with NGSO:50Eu^3+^ sub-MCs is 0.018701, which is much lower than the LED fabricated with MCs (0.173283). It shows that the packaged LED devices filled with NGSO:Eu^3+^ sub-MCs have an excellent resistance to color casting.

**Figure 7:**
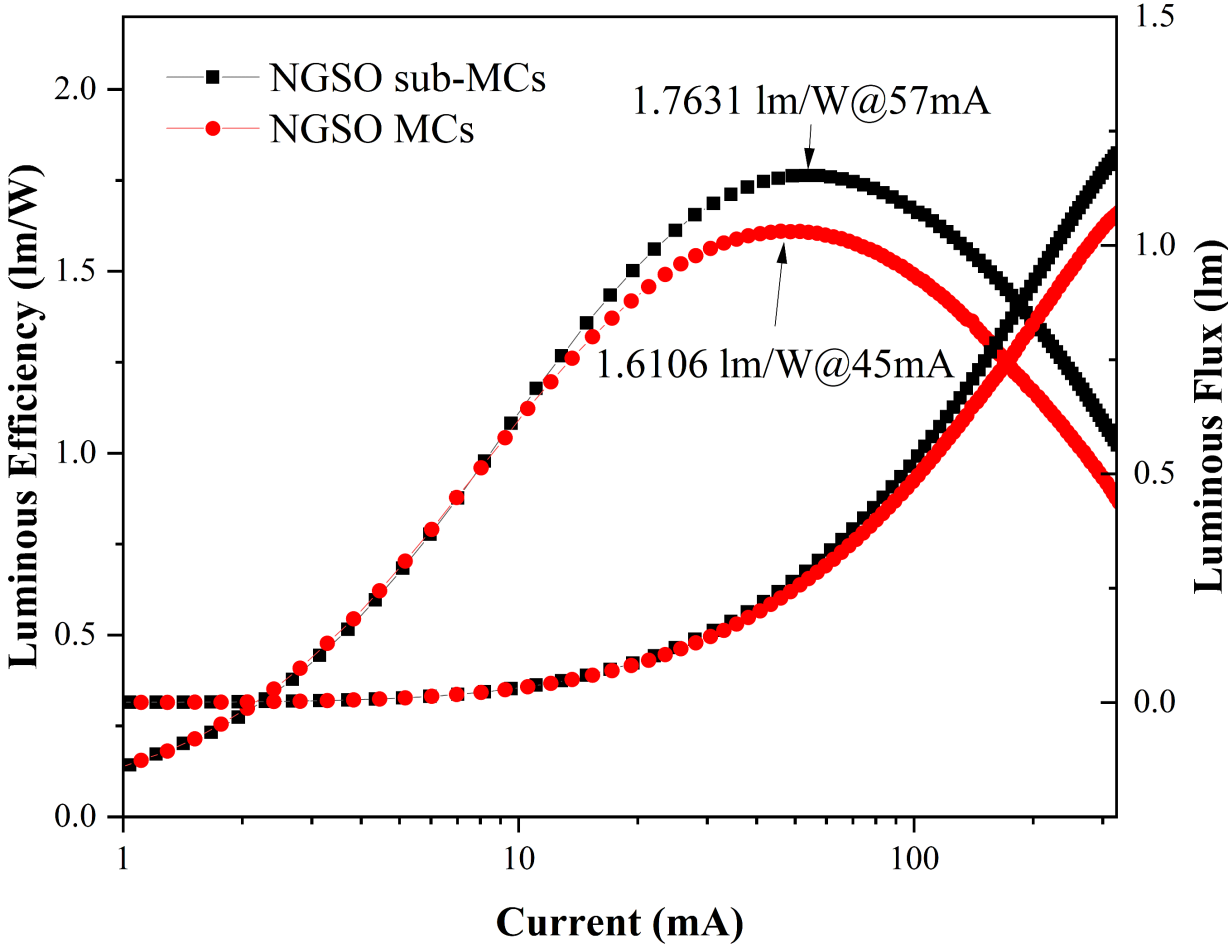
Luminous efficiency and luminous flux of NGSO:50Eu^3+^ sub-MCs and MCs.

The luminous efficiency and luminous flux of the two packaged LED devices were measured under various current intensities. Only the red light region of emissions was monitored. The volumes of phosphors filled in devices were almost the same. Although the PLQY of NGSO: Eu^3+^ sub-MCs was lower, its luminous efficiency was much higher. The luminous efficiency increased from 1.61 lm/W to 1.76 lm/W, an approximate 9.5% enhancement, as shown in Figure 7. The excitation wavelength was in the ultraviolet region. It is speculated that the smaller particle size will enhance the Rayleigh scattering, especially for the light of short wavelengths. For the phosphors with large particle sizes, for example, those crashed from bulk materials, Mie scattering might dominate. In that case, excitation light would mainly maintain the original propagating direction. The phosphors of smaller sizes might emit more light due to efficient irradiating by excitation light which is induced by scattering, even the LED was fabricated with a phosphor with a lower PLQY. Therefore, phosphors of smaller particle sizes, such as NGSO: Eu^3+^ sub-MCs, might have a higher luminous efficiency under the same excitation condition. That is what we observed in the experiments. Additionally, the luminous flux was also enhanced.

## 3. Materials and Methods

### 3.1. Chemicals

All reagents and solvents were used as received and without further purification. NaOH, oleic acid (OA), GdCl_3_·6H_2_O, EuCl_3_·6H_2_O, NH_4_F, tetraethyl orthosilicate (TEOS), Na_2_CO_3_, nano-sized SiO_2_ powder, and ammonia water (∽38 wt%) were purchased from Aladdin Reagent CO., Ltd., Shanghai, China. IGEPAL CO-520, Gd_2_O_3_, and Eu_2_O_3_ were purchased from Macklin Reagent CO., Ltd., Shanghai, China. Ethanol (EtOH) and cyclohexane were purchased from Sinopharm Chemical Reagent CO., Ltd., Shanghai, China. Deionized water was obtained from a Millipore IQ-7000 water purifier.

### 3.2. Synthesis of NaGdF_4_ Nanocrystals

NaGdF_4_ nanocrystals capped with OA ligands were synthesized via a modified solvothermal method based on our previous studies [34]. Typically, 0.4 g of NaOH, 5.34 g of OA, 6 mL of EtOH, 3 mL of deionized water, and 0.5 mmol RECl_3_ (RE = Gd, Eu) were mixed and stirred to form a homogeneous mixture, followed by the addition of 1 mL of NH_4_F aqueous solution (2 M). Once stirred for 30 min, the mixture was transferred into a 25 mL polytetrafluoroethylene (PTFE) autoclave and heated at 130 °C for 12 h. The products were obtained by precipitation and washed three times with cyclohexane and EtOH.

### 3.3. Synthesis of NaGdF_4_@SiO_2_ Core/Shell Nanocrystals

The reverse micelle method was used to coat the SiO_2_ layer on the NaGdF_4_ nanocrystals. First, 12 μmol of NaGdF_4_ nanocrystals and 1.1 g of CO-520 were well dispersed in 10 mL of cyclohexane. Then, 150 μL of ammonia and 100 μL of TEOS were added sequentially while stirring. Once stirred for 24 h, the product was precipitated by adding acetone and washed three times with EtOH, then dried at 70 °C overnight.

### 3.4. Synthesis of NaGd_9_Si_6_O_26_ Sub-MCs

Typically, the as-obtained NaGdF_4_@SiO_2_ core/shell nanoparticle powder was sintered at 400 °C for 2 h to remove residual organics, then the sintering temperature was raised to 1100 °C. Two hours later, the products were naturally cooled to room temperature. The as-obtained products were dispersed in the NaOH aqueous solution, transferred into a PTFE autoclave, and heated at 180 °C for 12 h. The final products were washed with deionized water and then dried at 70 °C overnight.

### 3.5. Synthesis of NaGd_9_Si_6_O_26_ MCs

NaGd_9_Si_6_O_26_ MCs were obtained using the high-temperature solid-state reaction. We weighed Na_2_CO_3_, SiO_2_, Gd_2_O_3_, and Eu_2_O_3_ according to the stoichiometric ratio, then we ground and mixed the compounds using an agate mortar, transferred them to a corundum crucible, and sintered them at 800 °C for 5 h to make sure that sodium carbonate was completely decomposed. Then, we raised the sintering temperature to 1400 °C for 10 h. Then, we fully ground the final product using an agate mortar for subsequent testing.

### 3.6. Characterizations

X-ray diffraction (XRD) patterns were acquired using a powder X-ray diffractometer (TD-3700, from Dandong Tongda Technology Co., Dandong, China) equipped with an X-ray tube with Cu target, operated under 30 kV. The images of the morphology of the samples were obtained using a high-resolution transmission electron microscope (TEM, Tecnai G2-20, FEI Co., Hillsboro, OR, USA) and a scanning electron microscope (SEM, Sigma300 from ZEISS, Oberkochen, Germany). Energy-dispersive X-ray spectroscopy (EDS) was used to obtain the content and distribution of the elements. Differential scanning calorimetry (DSC) and the thermogravimetrics (TGs) were measured using a synchronous thermal analyzer (STA 6000, from Perkin Elmer, Waltham, MA, USA). The sample was placed in an alumina crucible and heated in an air atmosphere. The photoluminescence (PL) measurements, photoluminescence excitation (PLE) spectra, and PL lifetime were carried out on a fluorescence spectrometer with a 150 W Xenon lamp (FLUROMAX PLUS, from Horiba Instruments INC, Kyoto, Japan). The red-emission LEDs were fabricated using NGSO phosphor and commercial blue-emission chips (central emission wavelength at 395 nm). The luminous efficiency and lumen flux were measured using a KEITHLEY 2400 Source Measure Unit (from Tektronix Inc, Beaverton, OR, USA) and a fluorescence quantum efficiency measurement system with an integrated sphere (from Labsphere Photonics Company, North Sutton, NH, USA), with a voltage range from 1 to 3.6 V.

## 4. Conclusions

In summary, we designed and developed a novel synthetic route for obtaining size controllable NaGd_9_Si_6_O_26_ sub-MCs by sintering NaGdF_4_@SiO_2_ nanoparticles at different temperatures. Part of SiO_2_ acted as a reactant to form NGSO together with NaGdF_4_. The rest of SiO_2_ provided space for NGSO to migrate and agglomerate, and played a certain role in space-confinement, thus the migration rate was well controlled. Higher temperatures promoted the growth of NGSO sub-MCs, resulting in larger particle sizes. Additionally, Eu^3+^-activated NGSO synthesized by the high-temperature solid-state reaction also exhibited a high PLQY, up to 91.5%, which is the highest value in rare earth silicates, to the best of our knowledge. The sub-micron NGSO phosphor leads to more efficient light extraction and scattering times, enhances the luminous efficiency and luminous flux, and greatly weakens the color deviation.

## Data Availability

Not applicable.

## Supplementary Materials

The following supporting information can be downloaded at: https://www.mdpi.com/article/10.3390/molecules28104214/s1, Table S1: Atom ratio statistics of NGSO sub-MCs; Table S2: Lattice parameters of the NaGd_9_Si_6_O_26_ phase and NaY_9_Si_6_O_26_ phase; Table S3: Atom ratio statistics of NGSO:50Eu^3+^ sub-MCs; Table S4: Atom ratio statistics of NGSO:50Eu^3+^ MCs; Figure S1 Rietveld refinement of XRD results of NaGd_9_Si_6_O_26_ from a CIF file of NaY_9_Si_6_O_26_; Figure S2. XRD patterens of 50 mol% Eu^3+^ doped NaGd_9_Si_6_O_26_ (NGSO) sub-MCs and MCs; Figure S3. Scanning Electron Microscopes (SEM) images and Energy dispersive X-ray Spectroscopy (EDS) mapping of NGSO:50Eu^3+^ sub-MCs; Figure S4. EDS of NGSO:50Eu^3+^ sub-MCs; Figure S5. SEM images and EDS mapping of NGSO:50Eu^3+^ MCs; Figure S6. EDS of NGSO:50Eu^3+^ MCs; Figure S7. PLQY of NGSO:50Eu^3+^ sub-MCs; Figure S8. PLQY of NGSO:50Eu^3+^ MCs.
