# Supplementary material for: Synthesis of Eu3+-Doped NaGd9Si6O26 Sub-Microcrystals from a NaGdF4@SiO2 Structure
Source: Molecules. 2023 May 20;28(10):4214. doi: 10.3390/molecules28104214 (PMC10222598; doi:10.3390/molecules28104214)
Supplement: Supplementary file 1 [file molecules-28-04214-s001.zip › molecules-2365410-supplementary.pdf]

Supplemental document

Table S1 Atom ratio statistics of NGSO sub-MCs

Element (Line)	Weight%	Error%	Atom%	Error%
C (K)	67.42	±0.20	82.07	±0.25
O (K)	17.01	±0.22	15.54	±0.20
Na (K)	0.38	±0.06	0.24	±0.04
Si (K)	1.64	±0.08	0.86	±0.04
Gd (M)	13.54	±0.50	1.29	±0.05
Total	100.00		100.00	

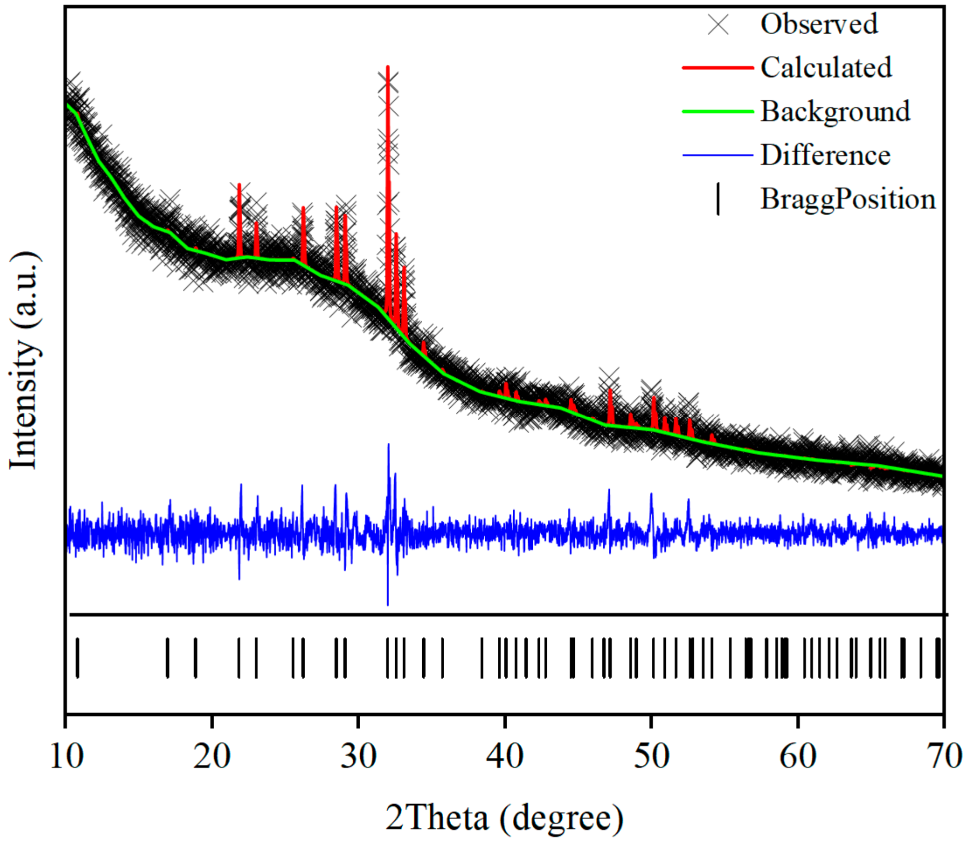

Figure S1 Rietveld refinement of XRD results of $NaGd_9Si_6O_{26}$ from a CIF file of $NaY_9Si_6O_{26}$

Table S2 Lattice parameters of the $NaGd_9Si_6O_{26}$ phase and $NaY_9Si_6O_{26}$ phase

Molecular Formula	$NaGd_9Si_6O_{26}$	$NaY_9Si_6O_{26}$
Molecular Weight	2022.74 g/mol	1407.64 g/mol
Crystal System	Hexagonal	Hexagonal
Space Group	P 63/m	P 63/m
a	9.361 Å	9.334 Å
b	9.361 Å	9.334 Å
c	6.809 Å	6.759 Å
α	90°	90°
β	90°	90°
γ	120°	120°
Cell Volume	516.84 $Å^3$	509.98 $Å^3$
Density	5.4103 g/cm^3	4.583 g/cm^3

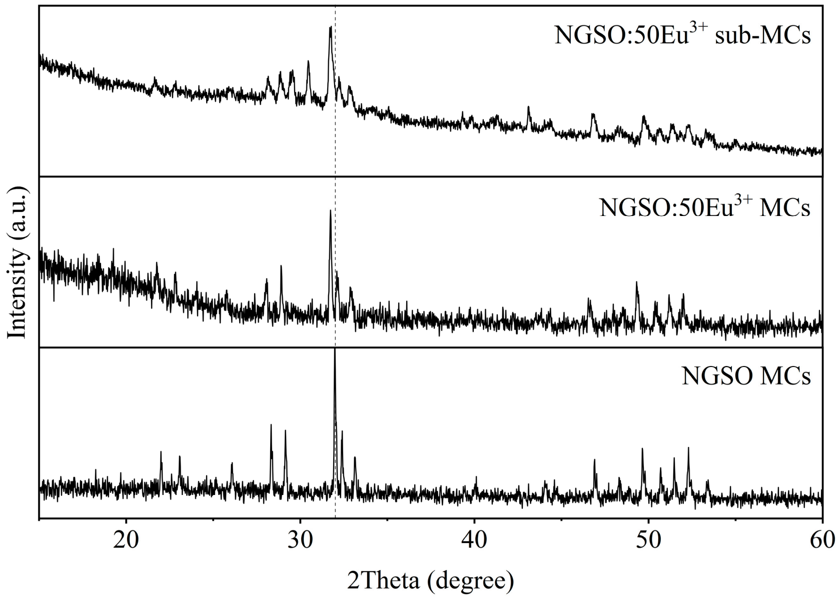

Figure S2. XRD patterens of 50mol% Eu^{3+} doped $NaGd_9Si_6O_{26}$ (NGSO) sub-MCs and MCs

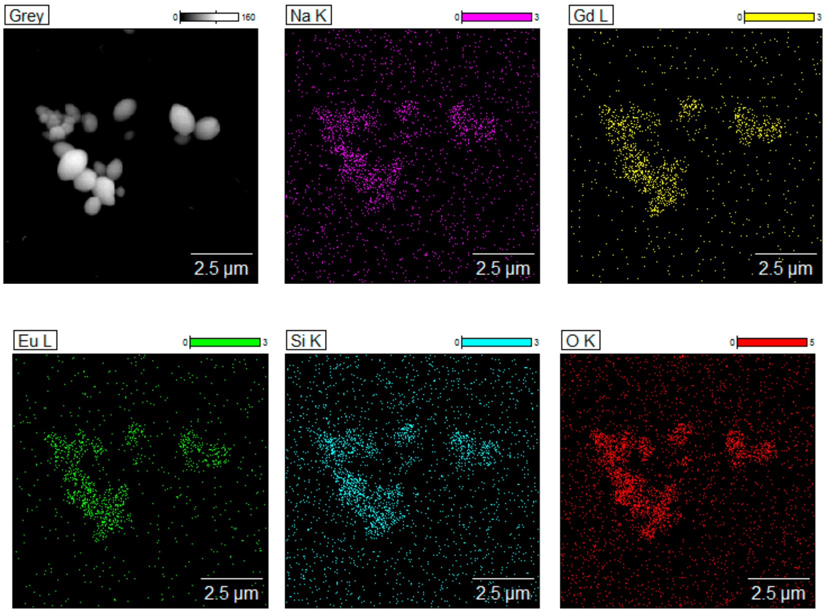

Figure S3. Scanning Electron Microscopes (SEM) images and Energy dispersive X-Ray Spectroscopy (EDS) mapping of NGSO:50Eu^{3+} sub-MCs

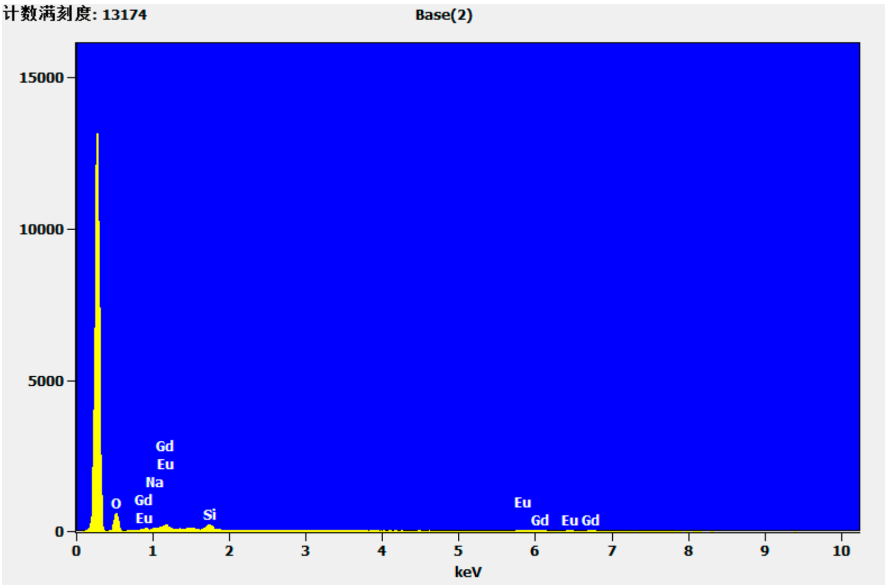

Figure S4. EDS of NGSO:50Eu^{3+} sub-MCs

Table S3 Atom ratio statistics of NGSO:50Eu^{3+} sub-MCs

Element (Line)	Weight%	Error%	Atom%	Error%
O (K)	19.52	±0.55	63.45	±1.77
Na (K)	1.05	±0.19	2.37	±0.43
Si (K)	4.91	±0.33	9.09	±0.62
Eu (M)	39.37	±2.64	13.47	±0.90
Gd (M)	35.16	±4.36	11.63	±1.44
Total	100.00		100.00	

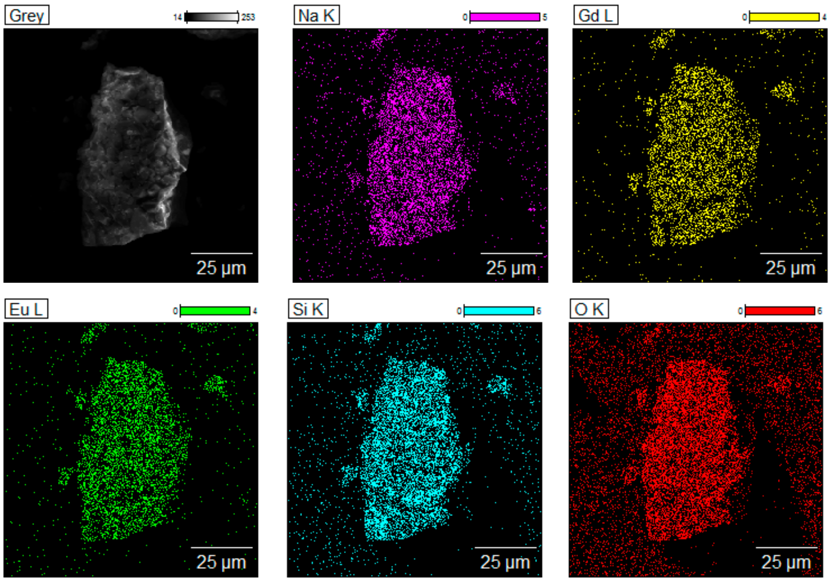

Figure S5.SEM images and EDS mapping of NGSO:50Eu^{3+} MCs

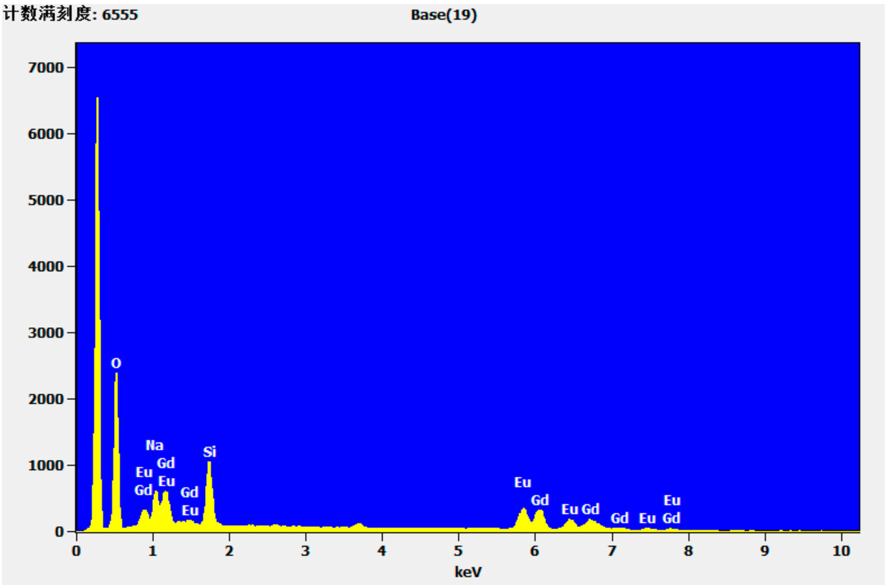

Figure S6. EDS of NGSO:50Eu^{3+} MCs

Table S4 Atom ratio statistics of NGSO:50Eu^{3+} MCs

Element (Line)	Weight%	Aberration%	Atom%	Aberration%
O (K)	37.99	±0.39	75.50	±0.78
Na (K)	0.96	±0.27	1.32	±0.38
Si (K)	11.46	±0.12	12.98	±0.14
Eu (M)	24.52	±1.10	5.13	±0.23
Gd (M)	25.07	±1.26	5.07	±0.26
Total	100.00		100.00	

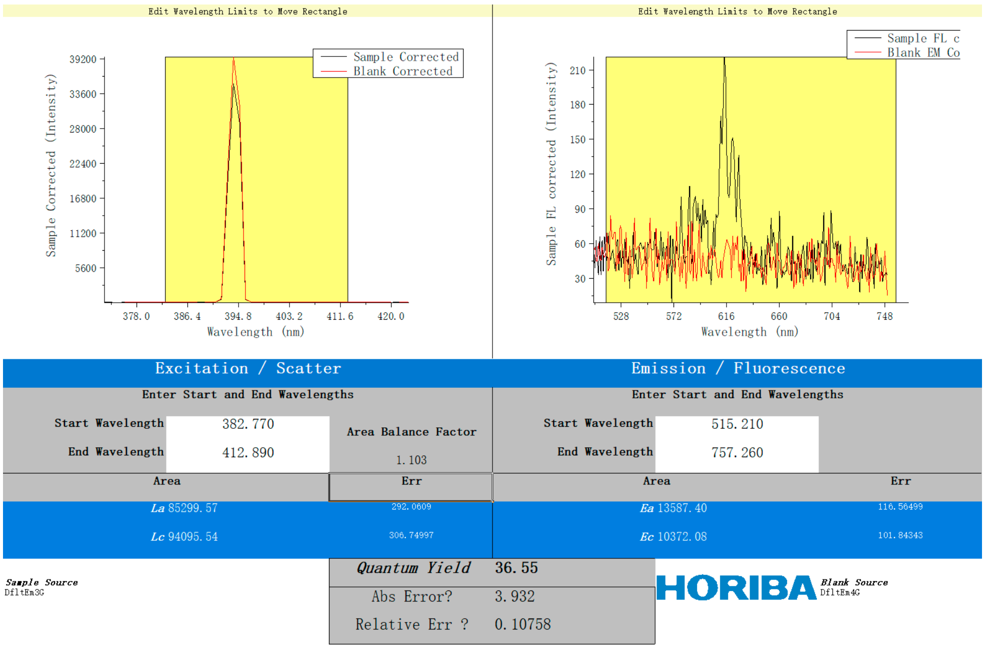

Figure S7. PLQY of NGSO:50Eu^{3+} sub-MCs

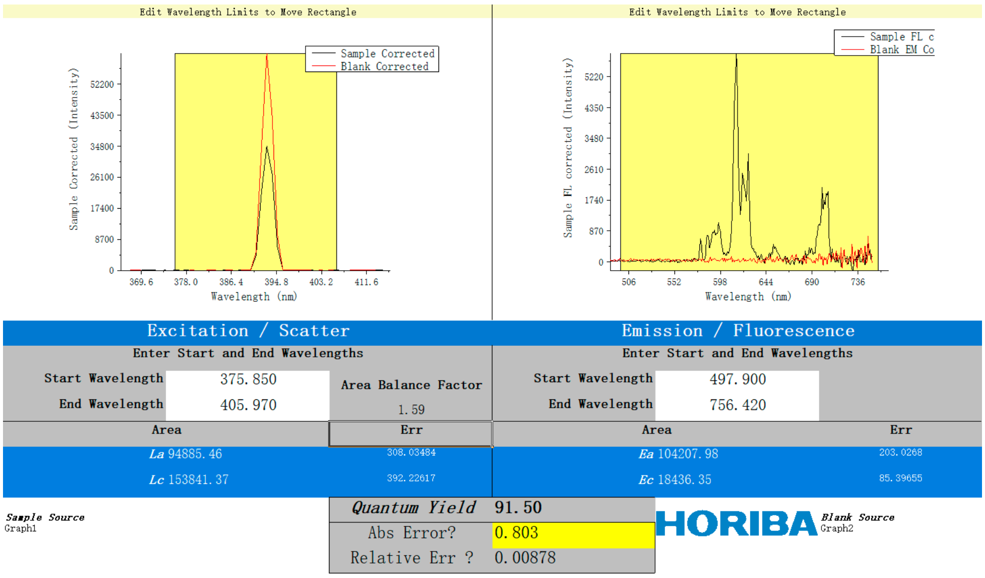

Figure S8. PLQY of NGSO:50Eu^{3+} MCs
